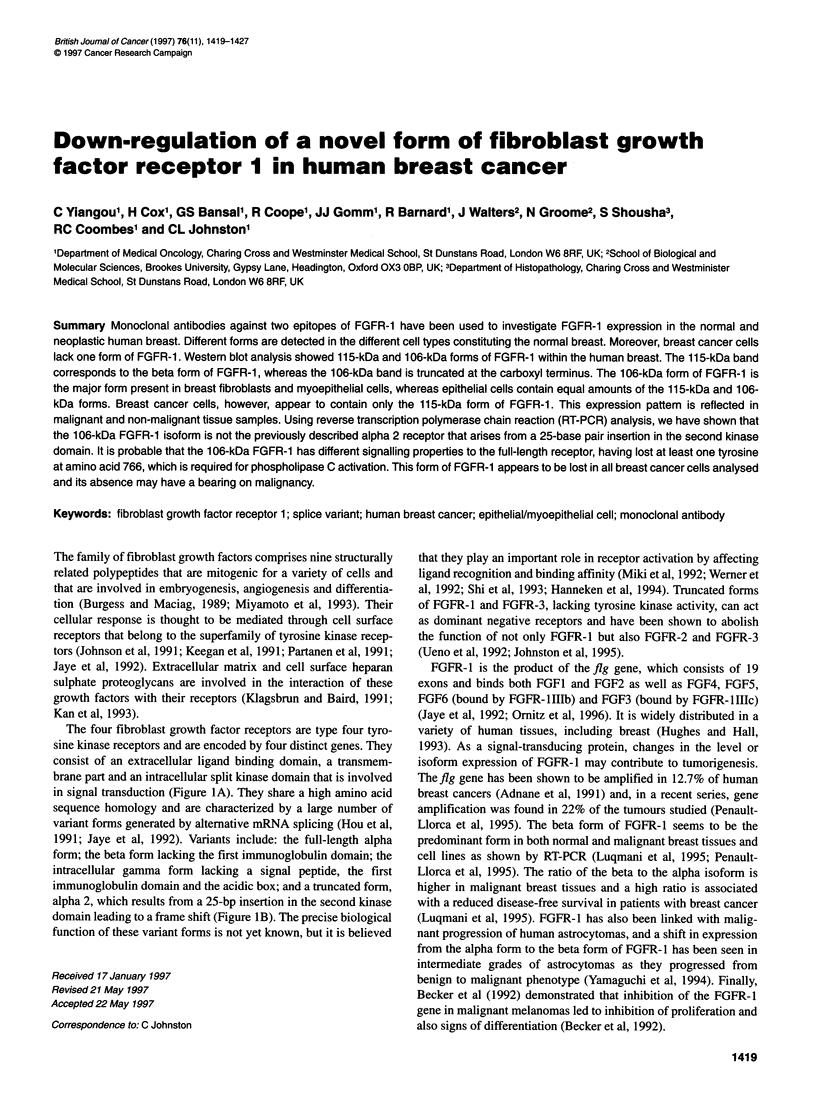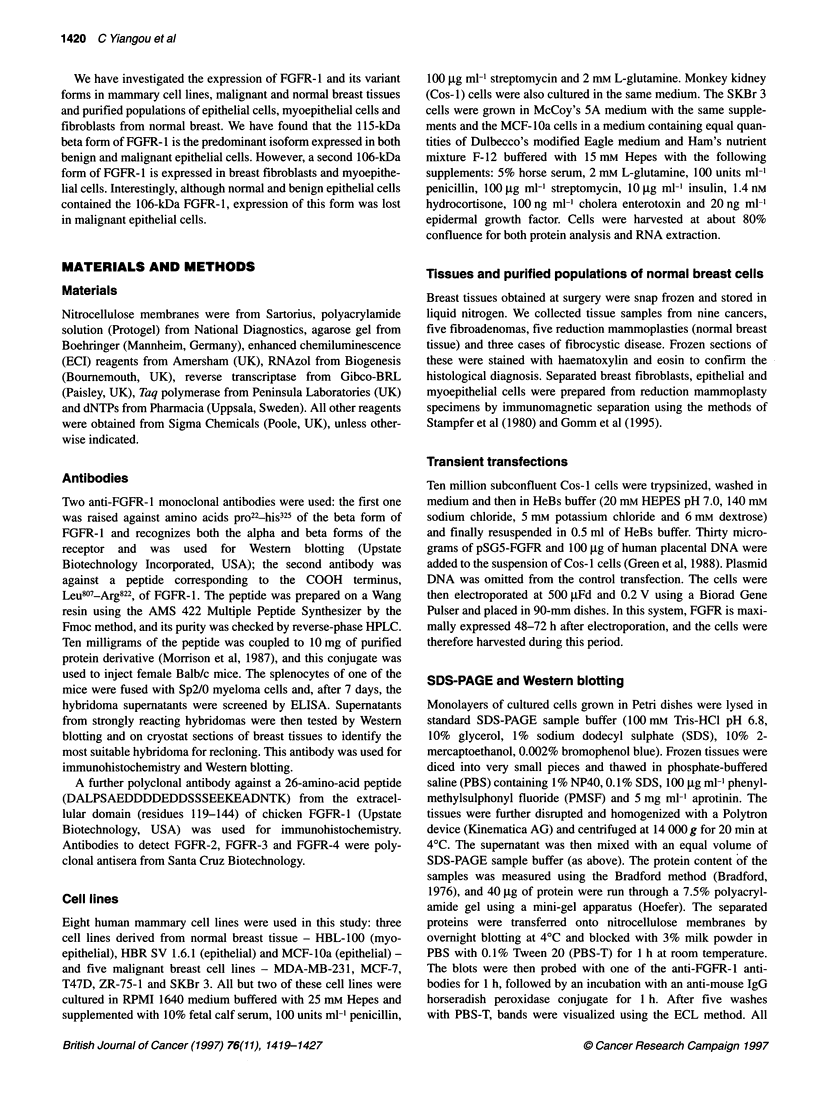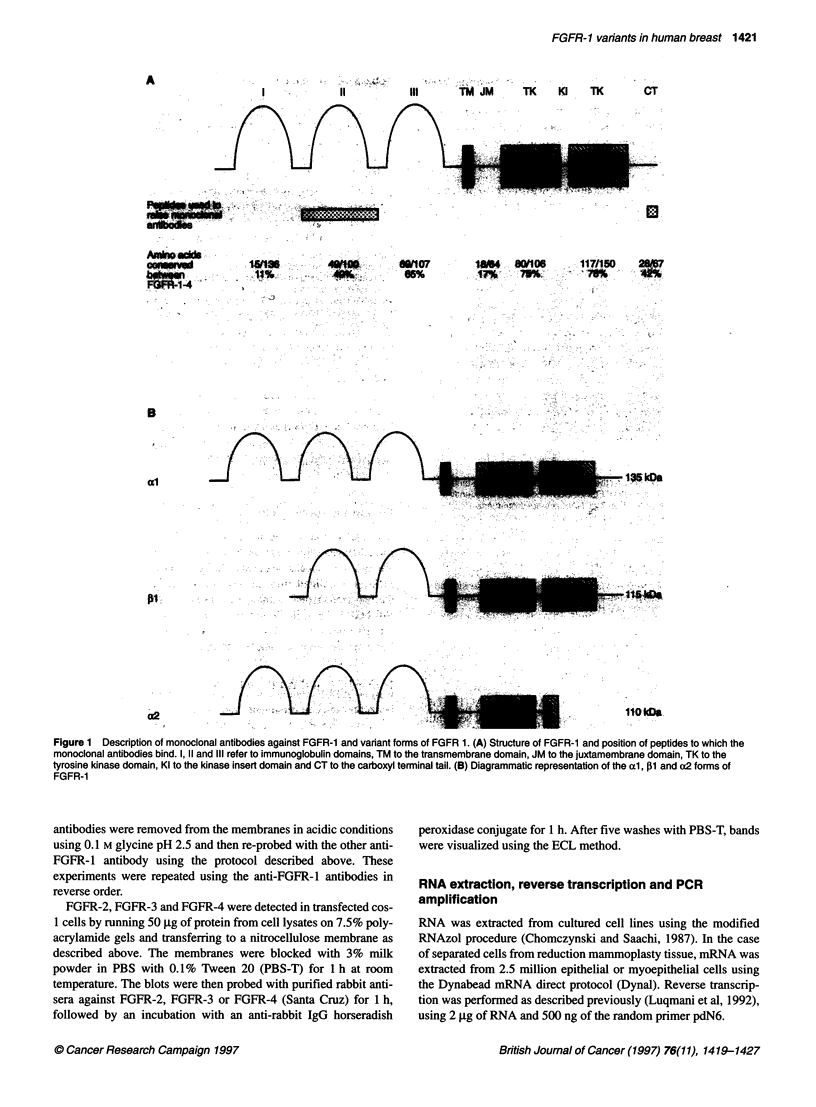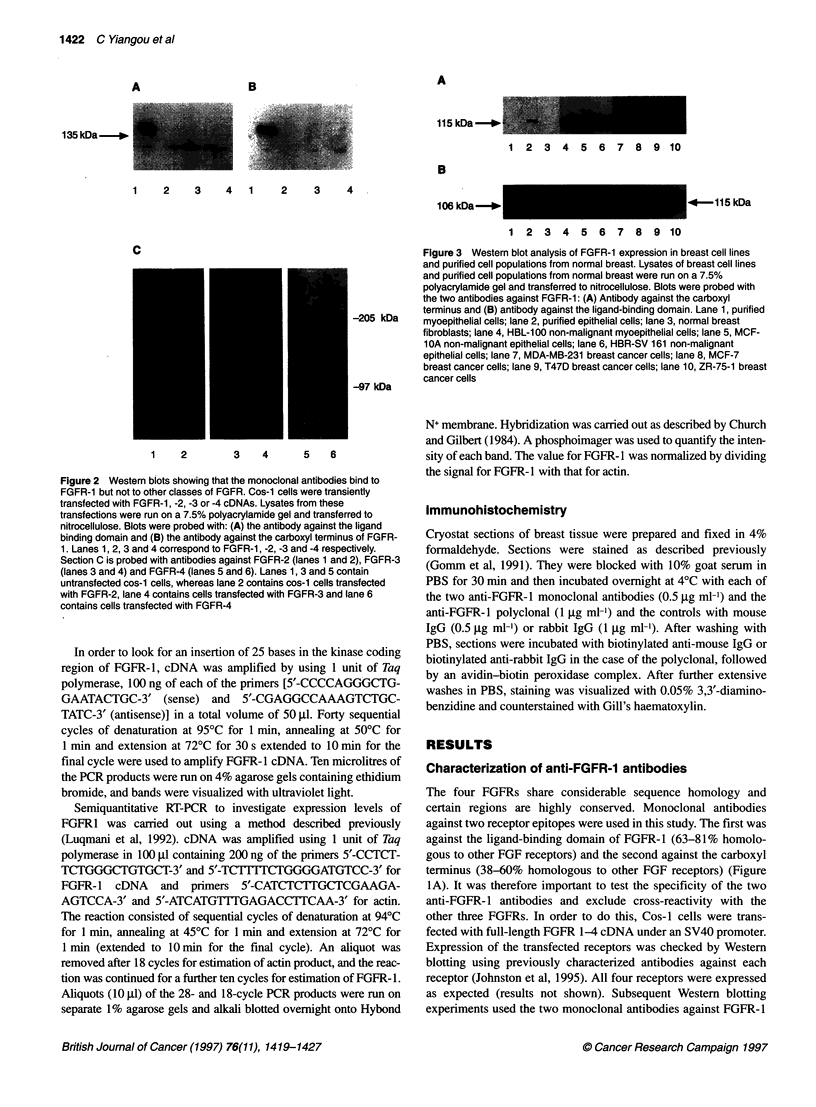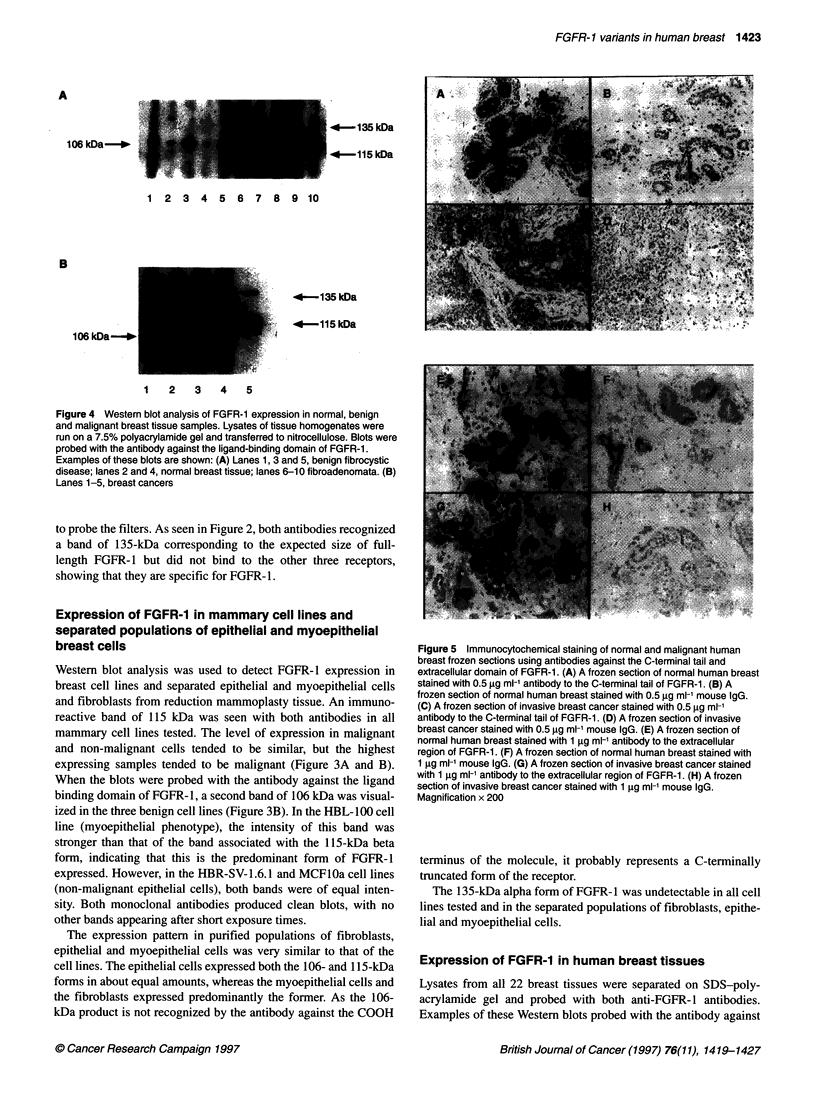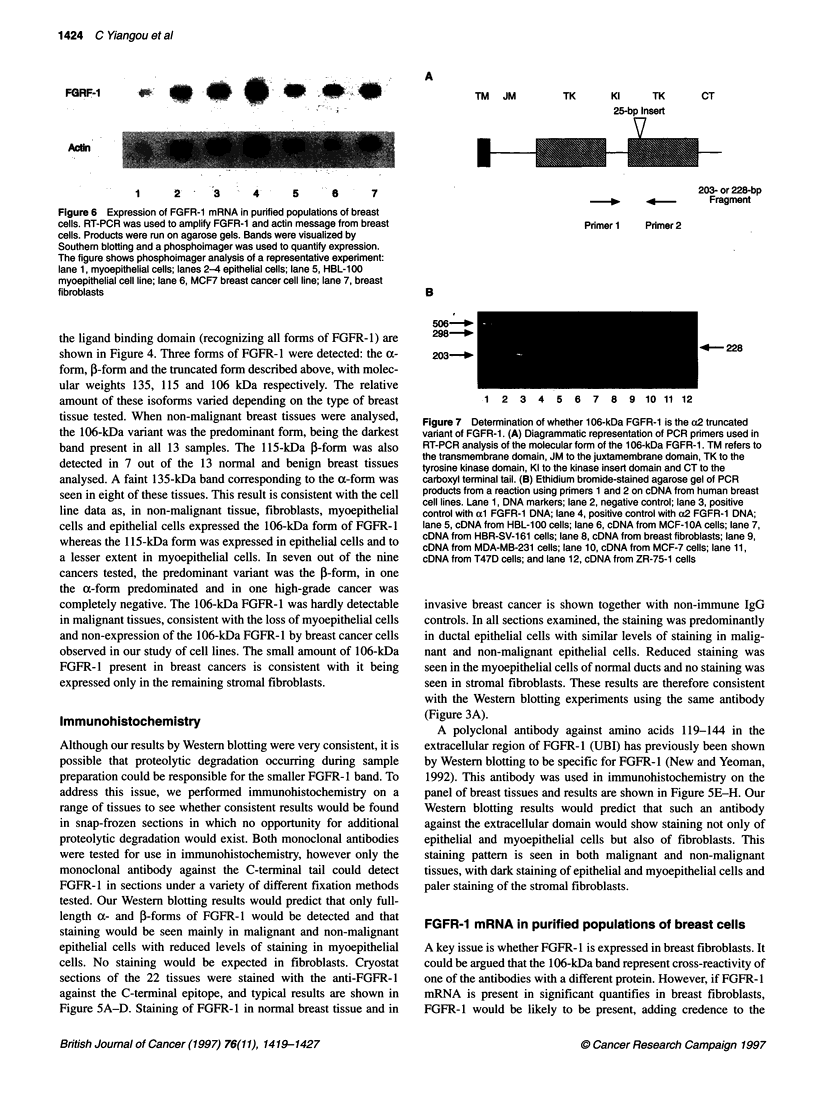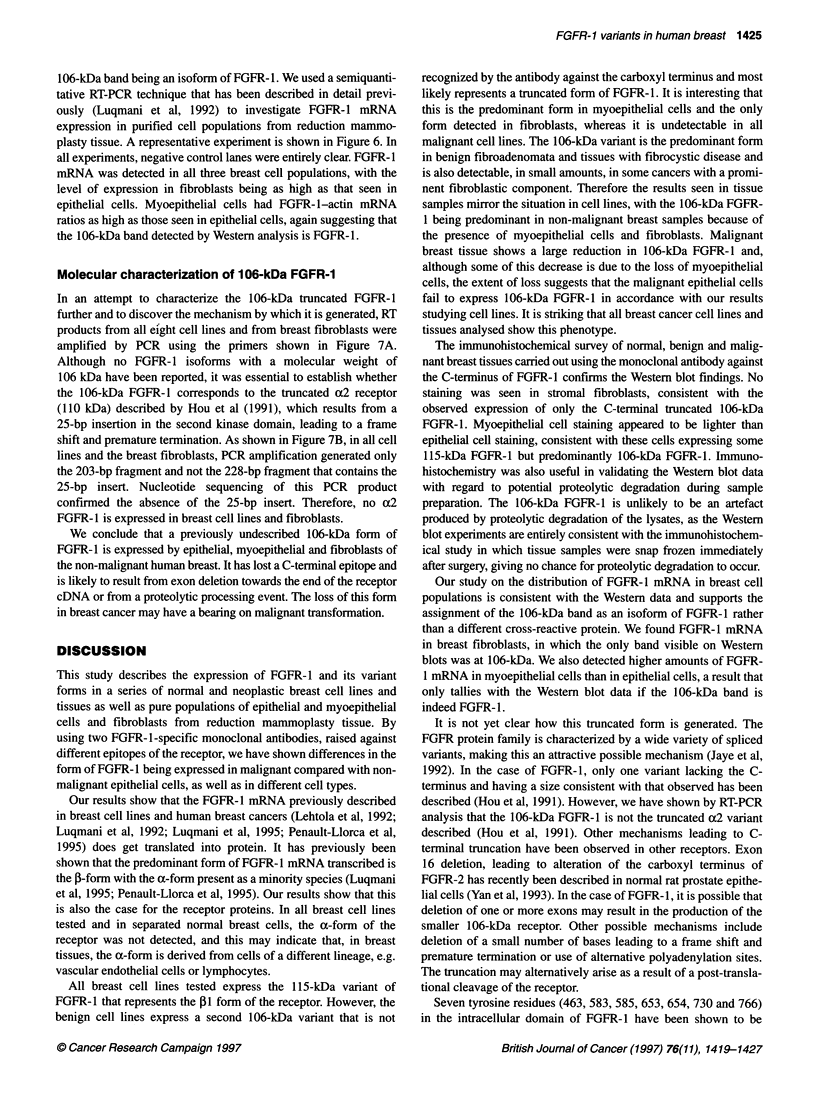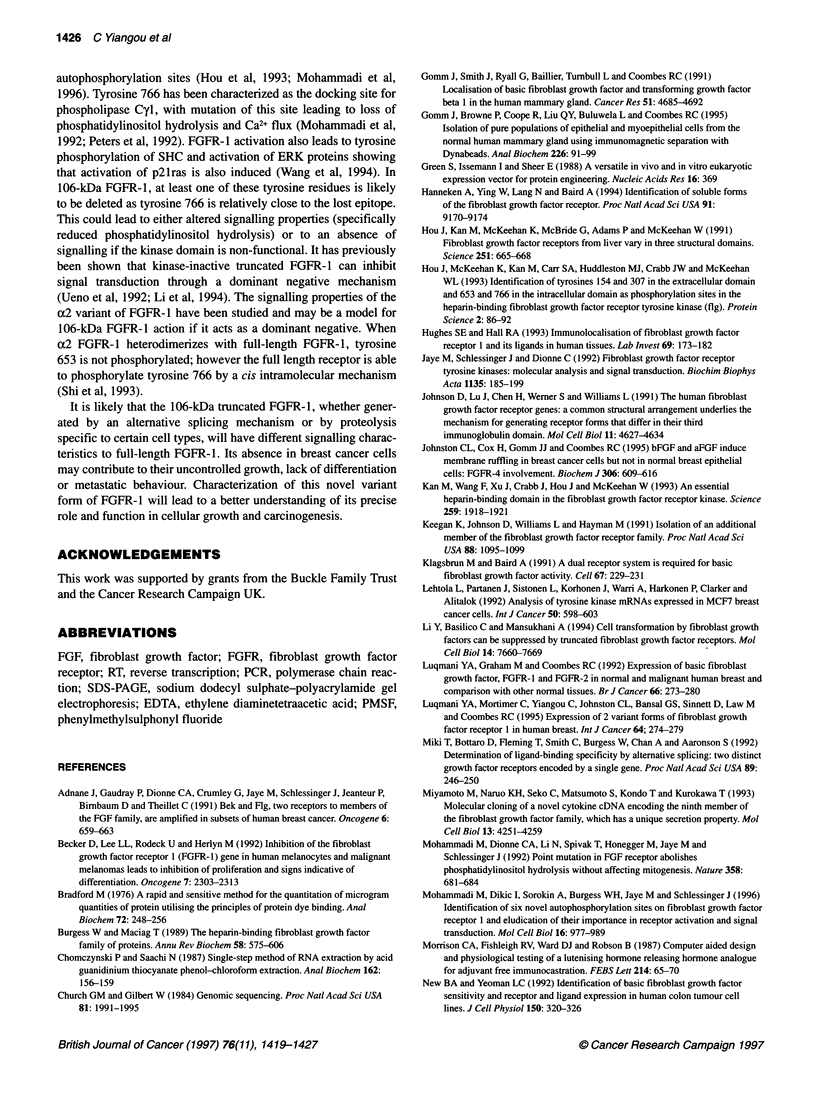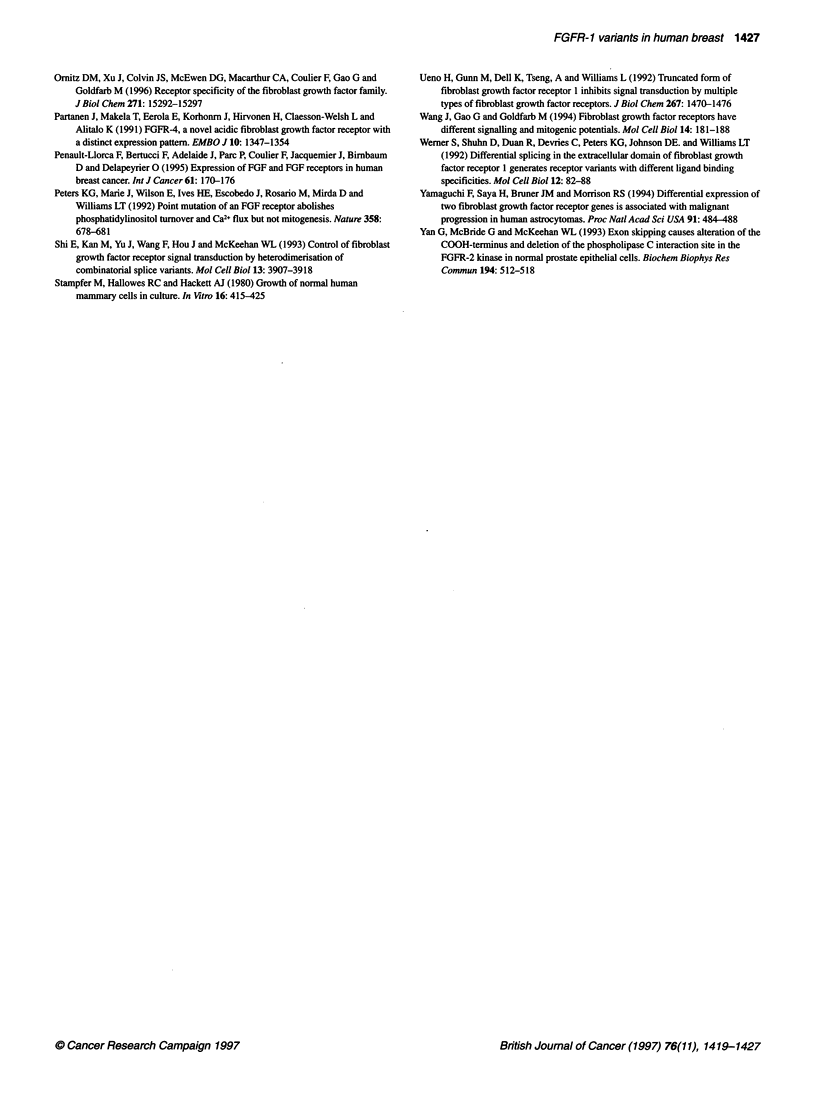# Down-regulation of a novel form of fibroblast growth factor receptor 1 in human breast cancer.

**DOI:** 10.1038/bjc.1997.573

**Published:** 1997

**Authors:** C. Yiangou, H. Cox, G. S. Bansal, R. Coope, J. J. Gomm, R. Barnard, J. Walters, N. Groome, S. Shousha, R. C. Coombes, C. L. Johnston

**Affiliations:** Department of Medical Oncology, Charing Cross and Westminster Medical School, London, UK.

## Abstract

**Images:**


British Journal of Cancer (1997) 76(11), 1419–1427
© 1997 Cancer Research Campaign

### Down-regulation of a novel form of fibroblast growth factor receptor 1 in human breast cancer

C Yiangou[1], H Cox[1], GS Bansal[1], R Coope[1], JJ Gomm[1], R Barnard[1], J Walters[2], N Groome[2], S Shousha[3], RC Coombes[1] and CL Johnston[1]

[1]Department of Medical Oncology, Charing Cross and Westminster Medical School, St Dunstans Road, London W6 8RF, UK; [2]School of Biological and Molecular Sciences, Brookes University, Gypsy Lane, Headington, Oxford OX3 0BP, UK; [3]Department of Histopathology, Charing Cross and Westminister Medical School, St Dunstans Road, London W6 8RF, UK

**Summary** Monoclonal antibodies against two epitopes of FGFR-1 have been used to investigate FGFR-1 expression in the normal and neoplastic human breast. Different forms are detected in the different cell types constituting the normal breast. Moreover, breast cancer cells lack one form of FGFR-1. Western blot analysis showed 115-kDa and 106-kDa forms of FGFR-1 within the human breast. The 115-kDa band corresponds to the beta form of FGFR-1, whereas the 106-kDa band is truncated at the carboxyl terminus. The 106-kDa form of FGFR-1 is the major form present in breast fibroblasts and myoepithelial cells, whereas epithelial cells contain equal amounts of the 115-kDa and 106-kDa forms. Breast cancer cells, however, appear to contain only the 115-kDa form of FGFR-1. This expression pattern is reflected in malignant and non-malignant tissue samples. Using reverse transcription polymerase chain reaction (RT-PCR) analysis, we have shown that the 106-kDa FGFR-1 isoform is not the previously described alpha 2 receptor that arises from a 25-base pair insertion in the second kinase domain. It is probable that the 106-kDa FGFR-1 has different signalling properties to the full-length receptor, having lost at least one tyrosine at amino acid 766, which is required for phospholipase C activation. This form of FGFR-1 appears to be lost in all breast cancer cells analysed and its absence may have a bearing on malignancy.

**Keywords:** fibroblast growth factor receptor 1; splice variant; human breast cancer; epithelial/myoepithelial cell; monoclonal antibody

The family of fibroblast growth factors comprises nine structurally related polypeptides that are mitogenic for a variety of cells and that are involved in embryogenesis, angiogenesis and differentiation (Burgess and Maciag, 1989; Miyamoto et al, 1993). Their cellular response is thought to be mediated through cell surface receptors that belong to the superfamily of tyrosine kinase receptors (Johnson et al, 1991; Keegan et al, 1991; Partanen et al, 1991; Jaye et al, 1992). Extracellular matrix and cell surface heparan sulphate proteoglycans are involved in the interaction of these growth factors with their receptors (Klagsbrun and Baird, 1991; Kan et al, 1993).

The four fibroblast growth factor receptors are type four tyrosine kinase receptors and are encoded by four distinct genes. They consist of an extracellular ligand binding domain, a transmembrane part and an intracellular split kinase domain that is involved in signal transduction (Figure 1A). They share a high amino acid sequence homology and are characterized by a large number of variant forms generated by alternative mRNA splicing (Hou et al, 1991; Jaye et al, 1992). Variants include: the full-length alpha form; the beta form lacking the first immunoglobulin domain; the intracellular gamma form lacking a signal peptide, the first immunoglobulin domain and the acidic box; and a truncated form, alpha 2, which results from a 25-bp insertion in the second kinase domain leading to a frame shift (Figure 1B). The precise biological function of these variant forms is not yet known, but it is believed that they play an important role in receptor activation by affecting ligand recognition and binding affinity (Miki et al, 1992; Werner et al, 1992; Shi et al, 1993; Hanneken et al, 1994). Truncated forms of FGFR-1 and FGFR-3, lacking tyrosine kinase activity, can act as dominant negative receptors and have been shown to abolish the function of not only FGFR-1 but also FGFR-2 and FGFR-3 (Ueno et al, 1992; Johnston et al, 1995).

FGFR-1 is the product of the *flg* gene, which consists of 19 exons and binds both FGF1 and FGF2 as well as FGF4, FGF5, FGF6 (bound by FGFR-1IIIb) and FGF3 (bound by FGFR-1IIIc) (Jaye et al, 1992; Ornitz et al, 1996). It is widely distributed in a variety of human tissues, including breast (Hughes and Hall, 1993). As a signal-transducing protein, changes in the level or isoform expression of FGFR-1 may contribute to tumorigenesis. The *flg* gene has been shown to be amplified in 12.7% of human breast cancers (Adnane et al, 1991) and, in a recent series, gene amplification was found in 22% of the tumours studied (Penault-Llorca et al, 1995). The beta form of FGFR-1 seems to be the predominant form in both normal and malignant breast tissues and cell lines as shown by RT-PCR (Luqmani et al, 1995; Penault-Llorca et al, 1995). The ratio of the beta to the alpha isoform is higher in malignant breast tissues and a high ratio is associated with a reduced disease-free survival in patients with breast cancer (Luqmani et al, 1995). FGFR-1 has also been linked with malignant progression of human astrocytomas, and a shift in expression from the alpha form to the beta form of FGFR-1 has been seen in intermediate grades of astrocytomas as they progressed from benign to malignant phenotype (Yamaguchi et al, 1994). Finally, Becker et al (1992) demonstrated that inhibition of the FGFR-1 gene in malignant melanomas led to inhibition of proliferation and also signs of differentiation (Becker et al, 1992).

*Received 17 January 1997*
*Revised 21 May 1997*
*Accepted 22 May 1997*

*Correspondence to:* C Johnston

We have investigated the expression of FGFR-1 and its variant forms in mammary cell lines, malignant and normal breast tissues and purified populations of epithelial cells, myoepithelial cells and fibroblasts from normal breast. We have found that the 115-kDa beta form of FGFR-1 is the predominant isoform expressed in both benign and malignant epithelial cells. However, a second 106-kDa form of FGFR-1 is expressed in breast fibroblasts and myoepithelial cells. Interestingly, although normal and benign epithelial cells contained the 106-kDa FGFR-1, expression of this form was lost in malignant epithelial cells.

### MATERIALS AND METHODS

### Materials

Nitrocellulose membranes were from Sartorius, polyacrylamide solution (Protogel) from National Diagnostics, agarose gel from Boehringer (Mannheim, Germany), enhanced chemiluminescence (ECl) reagents from Amersham (UK), RNAzol from Biogenesis (Bournemouth, UK), reverse transcriptase from Gibco-BRL (Paisley, UK), *Taq* polymerase from Peninsula Laboratories (UK) and dNTPs from Pharmacia (Uppsala, Sweden). All other reagents were obtained from Sigma Chemicals (Poole, UK), unless otherwise indicated.

### Antibodies

Two anti-FGFR-1 monoclonal antibodies were used: the first one was raised against amino acids $pro^{22}$–$his^{325}$ of the beta form of FGFR-1 and recognizes both the alpha and beta forms of the receptor and was used for Western blotting (Upstate Biotechnology Incorporated, USA); the second antibody was against a peptide corresponding to the COOH terminus, $Leu^{807}$–$Arg^{822}$, of FGFR-1. The peptide was prepared on a Wang resin using the AMS 422 Multiple Peptide Synthesizer by the Fmoc method, and its purity was checked by reverse-phase HPLC. Ten milligrams of the peptide was coupled to 10 mg of purified protein derivative (Morrison et al, 1987), and this conjugate was used to inject female Balb/c mice. The splenocytes of one of the mice were fused with Sp2/0 myeloma cells and, after 7 days, the hybridoma supernatants were screened by ELISA. Supernatants from strongly reacting hybridomas were then tested by Western blotting and on cryostat sections of breast tissues to identify the most suitable hybridoma for recloning. This antibody was used for immunohistochemistry and Western blotting.

A further polyclonal antibody against a 26-amino-acid peptide (DALPSAEDDDDEDDSSSEEKEADNTK) from the extracellular domain (residues 119–144) of chicken FGFR-1 (Upstate Biotechnology, USA) was used for immunohistochemistry. Antibodies to detect FGFR-2, FGFR-3 and FGFR-4 were polyclonal antisera from Santa Cruz Biotechnology.

### Cell lines

Eight human mammary cell lines were used in this study: three cell lines derived from normal breast tissue – HBL-100 (myoepithelial), HBR SV 1.6.1 (epithelial) and MCF-10a (epithelial) – and five malignant breast cell lines – MDA-MB-231, MCF-7, T47D, ZR-75-1 and SKBr 3. All but two of these cell lines were cultured in RPMI 1640 medium buffered with 25 mM Hepes and supplemented with 10% fetal calf serum, 100 units $ml^{-1}$ penicillin, 100 μg $ml^{-1}$ streptomycin and 2 mM L-glutamine. Monkey kidney (Cos-1) cells were also cultured in the same medium. The SKBr 3 cells were grown in McCoy's 5A medium with the same supplements and the MCF-10a cells in a medium containing equal quantities of Dulbecco's modified Eagle medium and Ham's nutrient mixture F-12 buffered with 15 mM Hepes with the following supplements: 5% horse serum, 2 mM L-glutamine, 100 units $ml^{-1}$ penicillin, 100 μg $ml^{-1}$ streptomycin, 10 μg $ml^{-1}$ insulin, 1.4 nM hydrocortisone, 100 ng $ml^{-1}$ cholera enterotoxin and 20 ng $ml^{-1}$ epidermal growth factor. Cells were harvested at about 80% confluence for both protein analysis and RNA extraction.

### Tissues and purified populations of normal breast cells

Breast tissues obtained at surgery were snap frozen and stored in liquid nitrogen. We collected tissue samples from nine cancers, five fibroadenomas, five reduction mammoplasties (normal breast tissue) and three cases of fibrocystic disease. Frozen sections of these were stained with haematoxylin and eosin to confirm the histological diagnosis. Separated breast fibroblasts, epithelial and myoepithelial cells were prepared from reduction mammoplasty specimens by immunomagnetic separation using the methods of Stampfer et al (1980) and Gomm et al (1995).

### Transient transfections

Ten million subconfluent Cos-1 cells were trypsinized, washed in medium and then in HeBs buffer (20 mM HEPES pH 7.0, 140 mM sodium chloride, 5 mM potassium chloride and 6 mM dextrose) and finally resuspended in 0.5 ml of HeBs buffer. Thirty micrograms of pSG5-FGFR and 100 μg of human placental DNA were added to the suspension of Cos-1 cells (Green et al, 1988). Plasmid DNA was omitted from the control transfection. The cells were then electroporated at 500 μFd and 0.2 V using a Biorad Gene Pulser and placed in 90-mm dishes. In this system, FGFR is maximally expressed 48–72 h after electroporation, and the cells were therefore harvested during this period.

### SDS-PAGE and Western blotting

Monolayers of cultured cells grown in Petri dishes were lysed in standard SDS-PAGE sample buffer (100 mM Tris-HCl pH 6.8, 10% glycerol, 1% sodium dodecyl sulphate (SDS), 10% 2-mercaptoethanol, 0.002% bromophenol blue). Frozen tissues were diced into very small pieces and thawed in phosphate-buffered saline (PBS) containing 1% NP40, 0.1% SDS, 100 μg $ml^{-1}$ phenylmethylsulphonyl fluoride (PMSF) and 5 mg $ml^{-1}$ aprotinin. The tissues were further disrupted and homogenized with a Polytron device (Kinematica AG) and centrifuged at 14 000 ***g*** for 20 min at 4°C. The supernatant was then mixed with an equal volume of SDS-PAGE sample buffer (as above). The protein content of the samples was measured using the Bradford method (Bradford, 1976), and 40 μg of protein were run through a 7.5% polyacrylamide gel using a mini-gel apparatus (Hoefer). The separated proteins were transferred onto nitrocellulose membranes by overnight blotting at 4°C and blocked with 3% milk powder in PBS with 0.1% Tween 20 (PBS-T) for 1 h at room temperature. The blots were then probed with one of the anti-FGFR-1 antibodies for 1 h, followed by an incubation with an anti-mouse IgG horseradish peroxidase conjugate for 1 h. After five washes with PBS-T, bands were visualized using the ECL method. All

© Cancer Research Campaign 1997

**Figure 1** Description of monoclonal antibodies against FGFR-1 and variant forms of FGFR 1. (**A**) Structure of FGFR-1 and position of peptides to which the monoclonal antibodies bind. I, II and III refer to immunoglobulin domains, TM to the transmembrane domain, JM to the juxtamembrane domain, TK to the tyrosine kinase domain, KI to the kinase insert domain and CT to the carboxyl terminal tail. (**B**) Diagrammatic representation of the α1, β1 and α2 forms of FGFR-1

antibodies were removed from the membranes in acidic conditions using 0.1 M glycine pH 2.5 and then re-probed with the other anti-FGFR-1 antibody using the protocol described above. These experiments were repeated using the anti-FGFR-1 antibodies in reverse order.

FGFR-2, FGFR-3 and FGFR-4 were detected in transfected cos-1 cells by running 50 μg of protein from cell lysates on 7.5% polyacrylamide gels and transferring to a nitrocellulose membrane as described above. The membranes were blocked with 3% milk powder in PBS with 0.1% Tween 20 (PBS-T) for 1 h at room temperature. The blots were then probed with purified rabbit antisera against FGFR-2, FGFR-3 or FGFR-4 (Santa Cruz) for 1 h, followed by an incubation with an anti-rabbit IgG horseradish peroxidase conjugate for 1 h. After five washes with PBS-T, bands were visualized using the ECL method.

### RNA extraction, reverse transcription and PCR amplification

RNA was extracted from cultured cell lines using the modified RNAzol procedure (Chomczynski and Saachi, 1987). In the case of separated cells from reduction mammoplasty tissue, mRNA was extracted from 2.5 million epithelial or myoepithelial cells using the Dynabead mRNA direct protocol (Dynal). Reverse transcription was performed as described previously (Luqmani et al, 1992), using 2 μg of RNA and 500 ng of the random primer pdN6.

**Figure 2** Western blots showing that the monoclonal antibodies bind to FGFR-1 but not to other classes of FGFR. Cos-1 cells were transiently transfected with FGFR-1, -2, -3 or -4 cDNAs. Lysates from these transfections were run on a 7.5% polyacrylamide gel and transferred to nitrocellulose. Blots were probed with: (**A**) the antibody against the ligand binding domain and (**B**) the antibody against the carboxyl terminus of FGFR-1. Lanes 1, 2, 3 and 4 correspond to FGFR-1, -2, -3 and -4 respectively. Section C is probed with antibodies against FGFR-2 (lanes 1 and 2), FGFR-3 (lanes 3 and 4) and FGFR-4 (lanes 5 and 6). Lanes 1, 3 and 5 contain untransfected cos-1 cells, whereas lane 2 contains cos-1 cells transfected with FGFR-2, lane 4 contains cells transfected with FGFR-3 and lane 6 contains cells transfected with FGFR-4

In order to look for an insertion of 25 bases in the kinase coding region of FGFR-1, cDNA was amplified by using 1 unit of *Taq* polymerase, 100 ng of each of the primers [5′-CCCCAGGGCTG-GAATACTGC-3′ (sense) and 5′-CGAGGCCAAAGTCTGC-TATC-3′ (antisense)] in a total volume of 50 µl. Forty sequential cycles of denaturation at 95°C for 1 min, annealing at 50°C for 1 min and extension at 72°C for 30 s extended to 10 min for the final cycle were used to amplify FGFR-1 cDNA. Ten microlitres of the PCR products were run on 4% agarose gels containing ethidium bromide, and bands were visualized with ultraviolet light.

Semiquantitative RT-PCR to investigate expression levels of FGFR1 was carried out using a method described previously (Luqmani et al, 1992). cDNA was amplified using 1 unit of *Taq* polymerase in 100 µl containing 200 ng of the primers 5′-CCTCT-TCTGGGCTGTGCT-3′ and 5′-TCTTTTCTGGGGATGTCC-3′ for FGFR-1 cDNA and primers 5′-CATCTCTTGCTCGAAGA-AGTCCA-3′ and 5′-ATCATGTTTGAGACCTTCAA-3′ for actin. The reaction consisted of sequential cycles of denaturation at 94°C for 1 min, annealing at 45°C for 1 min and extension at 72°C for 1 min (extended to 10 min for the final cycle). An aliquot was removed after 18 cycles for estimation of actin product, and the reaction was continued for a further ten cycles for estimation of FGFR-1. Aliquots (10 µl) of the 28- and 18-cycle PCR products were run on separate 1% agarose gels and alkali blotted overnight onto Hybond

**Figure 3** Western blot analysis of FGFR-1 expression in breast cell lines and purified cell populations from normal breast. Lysates of breast cell lines and purified cell populations from normal breast were run on a 7.5% polyacrylamide gel and transferred to nitrocellulose. Blots were probed with the two antibodies against FGFR-1: (**A**) Antibody against the carboxyl terminus and (**B**) antibody against the ligand-binding domain. Lane 1, purified myoepithelial cells; lane 2, purified epithelial cells; lane 3, normal breast fibroblasts; lane 4, HBL-100 non-malignant myoepithelial cells; lane 5, MCF-10A non-malignant epithelial cells; lane 6, HBR-SV 161 non-malignant epithelial cells; lane 7, MDA-MB-231 breast cancer cells; lane 8, MCF-7 breast cancer cells; lane 9, T47D breast cancer cells; lane 10, ZR-75-1 breast cancer cells

$N^+$ membrane. Hybridization was carried out as described by Church and Gilbert (1984). A phosphoimager was used to quantify the intensity of each band. The value for FGFR-1 was normalized by dividing the signal for FGFR-1 with that for actin.

### Immunohistochemistry

Cryostat sections of breast tissue were prepared and fixed in 4% formaldehyde. Sections were stained as described previously (Gomm et al, 1991). They were blocked with 10% goat serum in PBS for 30 min and then incubated overnight at 4°C with each of the two anti-FGFR-1 monoclonal antibodies (0.5 µg $ml^{-1}$) and the anti-FGFR-1 polyclonal (1 µg $ml^{-1}$) and the controls with mouse IgG (0.5 µg $ml^{-1}$) or rabbit IgG (1 µg $ml^{-1}$). After washing with PBS, sections were incubated with biotinylated anti-mouse IgG or biotinylated anti-rabbit IgG in the case of the polyclonal, followed by an avidin–biotin peroxidase complex. After further extensive washes in PBS, staining was visualized with 0.05% 3,3′-diaminobenzidine and counterstained with Gill's haematoxylin.

### RESULTS

### Characterization of anti-FGFR-1 antibodies

The four FGFRs share considerable sequence homology and certain regions are highly conserved. Monoclonal antibodies against two receptor epitopes were used in this study. The first was against the ligand-binding domain of FGFR-1 (63–81% homologous to other FGF receptors) and the second against the carboxyl terminus (38–60% homologous to other FGF receptors) (Figure 1A). It was therefore important to test the specificity of the two anti-FGFR-1 antibodies and exclude cross-reactivity with the other three FGFRs. In order to do this, Cos-1 cells were transfected with full-length FGFR 1–4 cDNA under an SV40 promoter. Expression of the transfected receptors was checked by Western blotting using previously characterized antibodies against each receptor (Johnston et al, 1995). All four receptors were expressed as expected (results not shown). Subsequent Western blotting experiments used the two monoclonal antibodies against FGFR-1

© Cancer Research Campaign 1997

**Figure 4** Western blot analysis of FGFR-1 expression in normal, benign and malignant breast tissue samples. Lysates of tissue homogenates were run on a 7.5% polyacrylamide gel and transferred to nitrocellulose. Blots were probed with the antibody against the ligand-binding domain of FGFR-1. Examples of these blots are shown: (**A**) Lanes 1, 3 and 5, benign fibrocystic disease; lanes 2 and 4, normal breast tissue; lanes 6–10 fibroadenomata. (**B**) Lanes 1–5, breast cancers to probe the filters. As seen in Figure 2, both antibodies recognized a band of 135-kDa corresponding to the expected size of full-length FGFR-1 but did not bind to the other three receptors, showing that they are specific for FGFR-1.

### Expression of FGFR-1 in mammary cell lines and separated populations of epithelial and myoepithelial breast cells

Western blot analysis was used to detect FGFR-1 expression in breast cell lines and separated epithelial and myoepithelial cells and fibroblasts from reduction mammoplasty tissue. An immunoreactive band of 115 kDa was seen with both antibodies in all mammary cell lines tested. The level of expression in malignant and non-malignant cells tended to be similar, but the highest expressing samples tended to be malignant (Figure 3A and B). When the blots were probed with the antibody against the ligand binding domain of FGFR-1, a second band of 106 kDa was visualized in the three benign cell lines (Figure 3B). In the HBL-100 cell line (myoepithelial phenotype), the intensity of this band was stronger than that of the band associated with the 115-kDa beta form, indicating that this is the predominant form of FGFR-1 expressed. However, in the HBR-SV-1.6.1 and MCF10a cell lines (non-malignant epithelial cells), both bands were of equal intensity. Both monoclonal antibodies produced clean blots, with no other bands appearing after short exposure times.

The expression pattern in purified populations of fibroblasts, epithelial and myoepithelial cells was very similar to that of the cell lines. The epithelial cells expressed both the 106- and 115-kDa forms in about equal amounts, whereas the myoepithelial cells and the fibroblasts expressed predominantly the former. As the 106-kDa product is not recognized by the antibody against the COOH terminus of the molecule, it probably represents a C-terminally truncated form of the receptor.

**Figure 5** Immunocytochemical staining of normal and malignant human breast frozen sections using antibodies against the C-terminal tail and extracellular domain of FGFR-1. (**A**) A frozen section of normal human breast stained with 0.5 μg ml$^{-1}$ antibody to the C-terminal tail of FGFR-1. (**B**) A frozen section of normal human breast stained with 0.5 μg ml$^{-1}$ mouse IgG. (**C**) A frozen section of invasive breast cancer stained with 0.5 μg ml$^{-1}$ antibody to the C-terminal tail of FGFR-1. (**D**) A frozen section of invasive breast cancer stained with 0.5 μg ml$^{-1}$ mouse IgG. (**E**) A frozen section of normal human breast stained with 1 μg ml$^{-1}$ antibody to the extracellular region of FGFR-1. (**F**) A frozen section of normal human breast stained with 1 μg ml$^{-1}$ mouse IgG. (**G**) A frozen section of invasive breast cancer stained with 1 μg ml$^{-1}$ antibody to the extracellular region of FGFR-1. (**H**) A frozen section of invasive breast cancer stained with 1 μg ml$^{-1}$ mouse IgG. Magnification × 200

The 135-kDa alpha form of FGFR-1 was undetectable in all cell lines tested and in the separated populations of fibroblasts, epithelial and myoepithelial cells.

### Expression of FGFR-1 in human breast tissues

Lysates from all 22 breast tissues were separated on SDS–polyacrylamide gel and probed with both anti-FGFR-1 antibodies. Examples of these Western blots probed with the antibody against

**Figure 6** Expression of FGFR-1 mRNA in purified populations of breast cells. RT-PCR was used to amplify FGFR-1 and actin message from breast cells. Products were run on agarose gels. Bands were visualized by Southern blotting and a phosphoimager was used to quantify expression. The figure shows phosphoimager analysis of a representative experiment: lane 1, myoepithelial cells; lanes 2–4 epithelial cells; lane 5, HBL-100 myoepithelial cell line; lane 6, MCF7 breast cancer cell line; lane 7, breast fibroblasts

**Figure 7** Determination of whether 106-kDa FGFR-1 is the α2 truncated variant of FGFR-1. (**A**) Diagrammatic representation of PCR primers used in RT-PCR analysis of the molecular form of the 106-kDa FGFR-1. TM refers to the transmembrane domain, JM to the juxtamembrane domain, TK to the tyrosine kinase domain, KI to the kinase insert domain and CT to the carboxyl terminal tail. (**B**) Ethidium bromide-stained agarose gel of PCR products from a reaction using primers 1 and 2 on cDNA from human breast cell lines. Lane 1, DNA markers; lane 2, negative control; lane 3, positive control with α1 FGFR-1 DNA; lane 4, positive control with α2 FGFR-1 DNA; lane 5, cDNA from HBL-100 cells; lane 6, cDNA from MCF-10A cells; lane 7, cDNA from HBR-SV-161 cells; lane 8, cDNA from breast fibroblasts; lane 9, cDNA from MDA-MB-231 cells; lane 10, cDNA from MCF-7 cells; lane 11, cDNA from T47D cells; and lane 12, cDNA from ZR-75-1 cells the ligand binding domain (recognizing all forms of FGFR-1) are shown in Figure 4. Three forms of FGFR-1 were detected: the α-form, β-form and the truncated form described above, with molecular weights 135, 115 and 106 kDa respectively. The relative amount of these isoforms varied depending on the type of breast tissue tested. When non-malignant breast tissues were analysed, the 106-kDa variant was the predominant form, being the darkest band present in all 13 samples. The 115-kDa β-form was also detected in 7 out of the 13 normal and benign breast tissues analysed. A faint 135-kDa band corresponding to the α-form was seen in eight of these tissues. This result is consistent with the cell line data as, in non-malignant tissue, fibroblasts, myoepithelial cells and epithelial cells expressed the 106-kDa form of FGFR-1 whereas the 115-kDa form was expressed in epithelial cells and to a lesser extent in myoepithelial cells. In seven out of the nine cancers tested, the predominant variant was the β-form, in one the α-form predominated and in one high-grade cancer was completely negative. The 106-kDa FGFR-1 was hardly detectable in malignant tissues, consistent with the loss of myoepithelial cells and non-expression of the 106-kDa FGFR-1 by breast cancer cells observed in our study of cell lines. The small amount of 106-kDa FGFR-1 present in breast cancers is consistent with it being expressed only in the remaining stromal fibroblasts.

### Immunohistochemistry

Although our results by Western blotting were very consistent, it is possible that proteolytic degradation occurring during sample preparation could be responsible for the smaller FGFR-1 band. To address this issue, we performed immunohistochemistry on a range of tissues to see whether consistent results would be found in snap-frozen sections in which no opportunity for additional proteolytic degradation would exist. Both monoclonal antibodies were tested for use in immunohistochemistry, however only the monoclonal antibody against the C-terminal tail could detect FGFR-1 in sections under a variety of different fixation methods tested. Our Western blotting results would predict that only full-length α- and β-forms of FGFR-1 would be detected and that staining would be seen mainly in malignant and non-malignant epithelial cells with reduced levels of staining in myoepithelial cells. No staining would be expected in fibroblasts. Cryostat sections of the 22 tissues were stained with the anti-FGFR-1 against the C-terminal epitope, and typical results are shown in Figure 5A–D. Staining of FGFR-1 in normal breast tissue and in invasive breast cancer is shown together with non-immune IgG controls. In all sections examined, the staining was predominantly in ductal epithelial cells with similar levels of staining in malignant and non-malignant epithelial cells. Reduced staining was seen in the myoepithelial cells of normal ducts and no staining was seen in stromal fibroblasts. These results are therefore consistent with the Western blotting experiments using the same antibody (Figure 3A).

A polyclonal antibody against amino acids 119–144 in the extracellular region of FGFR-1 (UBI) has previously been shown by Western blotting to be specific for FGFR-1 (New and Yeoman, 1992). This antibody was used in immunohistochemistry on the panel of breast tissues and results are shown in Figure 5E–H. Our Western blotting results would predict that such an antibody against the extracellular domain would show staining not only of epithelial and myoepithelial cells but also of fibroblasts. This staining pattern is seen in both malignant and non-malignant tissues, with dark staining of epithelial and myoepithelial cells and paler staining of the stromal fibroblasts.

### FGFR-1 mRNA in purified populations of breast cells

A key issue is whether FGFR-1 is expressed in breast fibroblasts. It could be argued that the 106-kDa band represent cross-reactivity of one of the antibodies with a different protein. However, if FGFR-1 mRNA is present in significant quantifies in breast fibroblasts, FGFR-1 would be likely to be present, adding credence to the

 © Cancer Research Campaign 1997

106-kDa band being an isoform of FGFR-1. We used a semiquantitative RT-PCR technique that has been described in detail previously (Luqmani et al, 1992) to investigate FGFR-1 mRNA expression in purified cell populations from reduction mammoplasty tissue. A representative experiment is shown in Figure 6. In all experiments, negative control lanes were entirely clear. FGFR-1 mRNA was detected in all three breast cell populations, with the level of expression in fibroblasts being as high as that seen in epithelial cells. Myoepithelial cells had FGFR-1–actin mRNA ratios as high as those seen in epithelial cells, again suggesting that the 106-kDa band detected by Western analysis is FGFR-1.

### Molecular characterization of 106-kDa FGFR-1

In an attempt to characterize the 106-kDa truncated FGFR-1 further and to discover the mechanism by which it is generated, RT products from all eight cell lines and from breast fibroblasts were amplified by PCR using the primers shown in Figure 7A. Although no FGFR-1 isoforms with a molecular weight of 106 kDa have been reported, it was essential to establish whether the 106-kDa FGFR-1 corresponds to the truncated α2 receptor (110 kDa) described by Hou et al (1991), which results from a 25-bp insertion in the second kinase domain, leading to a frame shift and premature termination. As shown in Figure 7B, in all cell lines and the breast fibroblasts, PCR amplification generated only the 203-bp fragment and not the 228-bp fragment that contains the 25-bp insert. Nucleotide sequencing of this PCR product confirmed the absence of the 25-bp insert. Therefore, no α2 FGFR-1 is expressed in breast cell lines and fibroblasts.

We conclude that a previously undescribed 106-kDa form of FGFR-1 is expressed by epithelial, myoepithelial and fibroblasts of the non-malignant human breast. It has lost a C-terminal epitope and is likely to result from exon deletion towards the end of the receptor cDNA or from a proteolytic processing event. The loss of this form in breast cancer may have a bearing on malignant transformation.

### DISCUSSION

This study describes the expression of FGFR-1 and its variant forms in a series of normal and neoplastic breast cell lines and tissues as well as pure populations of epithelial and myoepithelial cells and fibroblasts from reduction mammoplasty tissue. By using two FGFR-1-specific monoclonal antibodies, raised against different epitopes of the receptor, we have shown differences in the form of FGFR-1 being expressed in malignant compared with non-malignant epithelial cells, as well as in different cell types.

Our results show that the FGFR-1 mRNA previously described in breast cell lines and human breast cancers (Lehtola et al, 1992; Luqmani et al, 1992; Luqmani et al, 1995; Penault-Llorca et al, 1995) does get translated into protein. It has previously been shown that the predominant form of FGFR-1 mRNA transcribed is the β-form with the α-form present as a minority species (Luqmani et al, 1995; Penault-Llorca et al, 1995). Our results show that this is also the case for the receptor proteins. In all breast cell lines tested and in separated normal breast cells, the α-form of the receptor was not detected, and this may indicate that, in breast tissues, the α-form is derived from cells of a different lineage, e.g. vascular endothelial cells or lymphocytes.

All breast cell lines tested express the 115-kDa variant of FGFR-1 that represents the β1 form of the receptor. However, the benign cell lines express a second 106-kDa variant that is not recognized by the antibody against the carboxyl terminus and most likely represents a truncated form of FGFR-1. It is interesting that this is the predominant form in myoepithelial cells and the only form detected in fibroblasts, whereas it is undetectable in all malignant cell lines. The 106-kDa variant is the predominant form in benign fibroadenomata and tissues with fibrocystic disease and is also detectable, in small amounts, in some cancers with a prominent fibroblastic component. Therefore the results seen in tissue samples mirror the situation in cell lines, with the 106-kDa FGFR-1 being predominant in non-malignant breast samples because of the presence of myoepithelial cells and fibroblasts. Malignant breast tissue shows a large reduction in 106-kDa FGFR-1 and, although some of this decrease is due to the loss of myoepithelial cells, the extent of loss suggests that the malignant epithelial cells fail to express 106-kDa FGFR-1 in accordance with our results studying cell lines. It is striking that all breast cancer cell lines and tissues analysed show this phenotype.

The immunohistochemical survey of normal, benign and malignant breast tissues carried out using the monoclonal antibody against the C-terminus of FGFR-1 confirms the Western blot findings. No staining was seen in stromal fibroblasts, consistent with the observed expression of only the C-terminal truncated 106-kDa FGFR-1. Myoepithelial cell staining appeared to be lighter than epithelial cell staining, consistent with these cells expressing some 115-kDa FGFR-1 but predominantly 106-kDa FGFR-1. Immunohistochemistry was also useful in validating the Western blot data with regard to potential proteolytic degradation during sample preparation. The 106-kDa FGFR-1 is unlikely to be an artefact produced by proteolytic degradation of the lysates, as the Western blot experiments are entirely consistent with the immunohistochemical study in which tissue samples were snap frozen immediately after surgery, giving no chance for proteolytic degradation to occur.

Our study on the distribution of FGFR-1 mRNA in breast cell populations is consistent with the Western data and supports the assignment of the 106-kDa band as an isoform of FGFR-1 rather than a different cross-reactive protein. We found FGFR-1 mRNA in breast fibroblasts, in which the only band visible on Western blots was at 106-kDa. We also detected higher amounts of FGFR-1 mRNA in myoepithelial cells than in epithelial cells, a result that only tallies with the Western blot data if the 106-kDa band is indeed FGFR-1.

It is not yet clear how this truncated form is generated. The FGFR protein family is characterized by a wide variety of spliced variants, making this an attractive possible mechanism (Jaye et al, 1992). In the case of FGFR-1, only one variant lacking the C-terminus and having a size consistent with that observed has been described (Hou et al, 1991). However, we have shown by RT-PCR analysis that the 106-kDa FGFR-1 is not the truncated α2 variant described (Hou et al, 1991). Other mechanisms leading to C-terminal truncation have been observed in other receptors. Exon 16 deletion, leading to alteration of the carboxyl terminus of FGFR-2 has recently been described in normal rat prostate epithelial cells (Yan et al, 1993). In the case of FGFR-1, it is possible that deletion of one or more exons may result in the production of the smaller 106-kDa receptor. Other possible mechanisms include deletion of a small number of bases leading to a frame shift and premature termination or use of alternative polyadenylation sites. The truncation may alternatively arise as a result of a post-translational cleavage of the receptor.

Seven tyrosine residues (463, 583, 585, 653, 654, 730 and 766) in the intracellular domain of FGFR-1 have been shown to be autophosphorylation sites (Hou et al, 1993; Mohammadi et al, 1996). Tyrosine 766 has been characterized as the docking site for phospholipase Cγ1, with mutation of this site leading to loss of phosphatidylinositol hydrolysis and $Ca^{2+}$ flux (Mohammadi et al, 1992; Peters et al, 1992). FGFR-1 activation also leads to tyrosine phosphorylation of SHC and activation of ERK proteins showing that activation of p21ras is also induced (Wang et al, 1994). In 106-kDa FGFR-1, at least one of these tyrosine residues is likely to be deleted as tyrosine 766 is relatively close to the lost epitope. This could lead to either altered signalling properties (specifically reduced phosphatidylinositol hydrolysis) or to an absence of signalling if the kinase domain is non-functional. It has previously been shown that kinase-inactive truncated FGFR-1 can inhibit signal transduction through a dominant negative mechanism (Ueno et al, 1992; Li et al, 1994). The signalling properties of the α2 variant of FGFR-1 have been studied and may be a model for 106-kDa FGFR-1 action if it acts as a dominant negative. When α2 FGFR-1 heterodimerizes with full-length FGFR-1, tyrosine 653 is not phosphorylated; however the full length receptor is able to phosphorylate tyrosine 766 by a *cis* intramolecular mechanism (Shi et al, 1993).

It is likely that the 106-kDa truncated FGFR-1, whether generated by an alternative splicing mechanism or by proteolysis specific to certain cell types, will have different signalling characteristics to full-length FGFR-1. Its absence in breast cancer cells may contribute to their uncontrolled growth, lack of differentiation or metastatic behaviour. Characterization of this novel variant form of FGFR-1 will lead to a better understanding of its precise role and function in cellular growth and carcinogenesis.

### ACKNOWLEDGEMENTS

This work was supported by grants from the Buckle Family Trust and the Cancer Research Campaign UK.

### ABBREVIATIONS

FGF, fibroblast growth factor; FGFR, fibroblast growth factor receptor; RT, reverse transcription; PCR, polymerase chain reaction; SDS-PAGE, sodium dodecyl sulphate–polyacrylamide gel electrophoresis; EDTA, ethylene diaminetetraacetic acid; PMSF, phenylmethylsulphonyl fluoride

### REFERENCES

Adnane J, Gaudray P, Dionne CA, Crumley G, Jaye M, Schlessinger J, Jeanteur P, Birnbaum D and Theillet C (1991) Bek and Flg, two receptors to members of the FGF family, are amplified in subsets of human breast cancer. *Oncogene* **6**: 659–663

Becker D, Lee LL, Rodeck U and Herlyn M (1992) Inhibition of the fibroblast growth factor receptor 1 (FGFR-1) gene in human melanocytes and malignant melanomas leads to inhibition of proliferation and signs indicative of differentiation. *Oncogene* **7**: 2303–2313

Bradford M (1976) A rapid and sensitive method for the quantitation of microgram quantities of protein utilising the principles of protein dye binding. *Anal Biochem* **72**: 248–256

Burgess W and Maciag T (1989) The heparin-binding fibroblast growth factor family of proteins. *Annu Rev Biochem* **58**: 575–606

Chomczynski P and Saachi N (1987) Single-step method of RNA extraction by acid guanidinium thiocyanate phenol–chloroform extraction. *Anal Biochem* **162**: 156–159

Church GM and Gilbert W (1984) Genomic sequencing. *Proc Natl Acad Sci USA* **81**: 1991–1995

Gomm J, Smith J, Ryall G, Baillier, Turnbull L and Coombes RC (1991) Localisation of basic fibroblast growth factor and transforming growth factor beta 1 in the human mammary gland. *Cancer Res* **51**: 4685–4692

Gomm J, Browne P, Coope R, Liu QY, Buluwela L and Coombes RC (1995) Isolation of pure populations of epithelial and myoepithelial cells from the normal human mammary gland using immunomagnetic separation with Dynabeads. *Anal Biochem* **226**: 91–99

Green S, Issemann I and Sheer E (1988) A versatile in vivo and in vitro eukaryotic expression vector for protein engineering. *Nucleic Acids Res* **16**: 369

Hanneken A, Ying W, Lang N and Baird A (1994) Identification of soluble forms of the fibroblast growth factor receptor. *Proc Natl Acad Sci USA* **91**: 9170–9174

Hou J, Kan M, McKeehan K, McBride G, Adams P and McKeehan W (1991) Fibroblast growth factor receptors from liver vary in three structural domains. *Science* **251**: 665–668

Hou J, McKeehan K, Kan M, Carr SA, Huddleston MJ, Crabb JW and McKeehan WL (1993) Identification of tyrosines 154 and 307 in the extracellular domain and 653 and 766 in the intracellular domain as phosphorylation sites in the heparin-binding fibroblast growth factor receptor tyrosine kinase (flg). *Protein Science* **2**: 86–92

Hughes SE and Hall RA (1993) Immunolocalisation of fibroblast growth factor receptor 1 and its ligands in human tissues. *Lab Invest* **69**: 173–182

Jaye M, Schlessinger J and Dionne C (1992) Fibroblast growth factor receptor tyrosine kinases: molecular analysis and signal transduction. *Biochim Biophys Acta* **1135**: 185–199

Johnson D, Lu J, Chen H, Werner S and Williams L (1991) The human fibroblast growth factor receptor genes: a common structural arrangement underlies the mechanism for generating receptor forms that differ in their third immunoglobulin domain. *Mol Cell Biol* **11**: 4627–4634

Johnston CL, Cox H, Gomm JJ and Coombes RC (1995) bFGF and aFGF induce membrane ruffling in breast cancer cells but not in normal breast epithelial cells: FGFR-4 involvement. *Biochem J* **306**: 609–616

Kan M, Wang F, Xu J, Crabb J, Hou J and McKeehan W (1993) An essential heparin-binding domain in the fibroblast growth factor receptor kinase. *Science* **259**: 1918–1921

Keegan K, Johnson D, Williams L and Hayman M (1991) Isolation of an additional member of the fibroblast growth factor receptor family. *Proc Natl Acad Sci USA* **88**: 1095–1099

Klagsbrun M and Baird A (1991) A dual receptor system is required for basic fibroblast growth factor activity. *Cell* **67**: 229–231

Lehtola L, Partanen J, Sistonen L, Korhonen J, Warri A, Harkonen P, Clarker and Alitalok (1992) Analysis of tyrosine kinase mRNAs expressed in MCF7 breast cancer cells. *Int J Cancer* **50**: 598–603

Li Y, Basilico C and Mansukhani A (1994) Cell transformation by fibroblast growth factors can be suppressed by truncated fibroblast growth factor receptors. *Mol Cell Biol* **14**: 7660–7669

Luqmani YA, Graham M and Coombes RC (1992) Expression of basic fibroblast growth factor, FGFR-1 and FGFR-2 in normal and malignant human breast and comparison with other normal tissues. *Br J Cancer* **66**: 273–280

Luqmani YA, Mortimer C, Yiangou C, Johnston CL, Bansal GS, Sinnett D, Law M and Coombes RC (1995) Expression of 2 variant forms of fibroblast growth factor receptor 1 in human breast. *Int J Cancer* **64**; 274–279

Miki T, Bottaro D, Fleming T, Smith C, Burgess W, Chan A and Aaronson S (1992) Determination of ligand-binding specificity by alternative splicing: two distinct growth factor receptors encoded by a single gene. *Proc Natl Acad Sci USA* **89**: 246–250

Miyamoto M, Naruo KH, Seko C, Matsumoto S, Kondo T and Kurokawa T (1993) Molecular cloning of a novel cytokine cDNA encoding the ninth member of the fibroblast growth factor family, which has a unique secretion property. *Mol Cell Biol* **13**: 4251–4259

Mohammadi M, Dionne CA, Li N, Spivak T, Honegger M, Jaye M and Schlessinger J (1992) Point mutation in FGF receptor abolishes phosphatidylinositol hydrolysis without affecting mitogenesis. *Nature* **358**: 681–684

Mohammadi M, Dikic I, Sorokin A, Burgess WH, Jaye M and Schlessinger J (1996) Identification of six novel autophosphorylation sites on fibroblast growth factor receptor 1 and eludication of their importance in receptor activation and signal transduction. *Mol Cell Biol* **16**: 977–989

Morrison CA, Fishleigh RV, Ward DJ and Robson B (1987) Computer aided design and physiological testing of a lutenising hormone releasing hormone analogue for adjuvant free immunocastration. *FEBS Lett* **214**: 65–70

New BA and Yeoman LC (1992) Identification of basic fibroblast growth factor sensitivity and receptor and ligand expression in human colon tumour cell lines. *J Cell Physiol* **150**: 320–326

 © Cancer Research Campaign 1997

Ornitz DM, Xu J, Colvin JS, McEwen DG, Macarthur CA, Coulier F, Gao G and Goldfarb M (1996) Receptor specificity of the fibroblast growth factor family. *J Biol Chem* **271**: 15292–15297

Partanen J, Makela T, Eerola E, Korhonrn J, Hirvonen H, Claesson-Welsh L and Alitalo K (1991) FGFR-4, a novel acidic fibroblast growth factor receptor with a distinct expression pattern. *EMBO J* **10**: 1347–1354

Penault-Llorca F, Bertucci F, Adelaide J, Parc P, Coulier F, Jacquemier J, Birnbaum D and Delapeyrier O (1995) Expression of FGF and FGF receptors in human breast cancer. *Int J Cancer* **61**: 170–176

Peters KG, Marie J, Wilson E, Ives HE, Escobedo J, Rosario M, Mirda D and Williams LT (1992) Point mutation of an FGF receptor abolishes phosphatidylinositol turnover and $Ca^{2+}$ flux but not mitogenesis. *Nature* **358**: 678–681

Shi E, Kan M, Yu J, Wang F, Hou J and McKeehan WL (1993) Control of fibroblast growth factor receptor signal transduction by heterodimerisation of combinatorial splice variants. *Mol Cell Biol* **13**: 3907–3918

Stampfer M, Hallowes RC and Hackett AJ (1980) Growth of normal human mammary cells in culture. *In Vitro* **16**: 415–425

Ueno H, Gunn M, Dell K, Tseng, A and Williams L (1992) Truncated form of fibroblast growth factor receptor 1 inhibits signal transduction by multiple types of fibroblast growth factor receptors. *J Biol Chem* **267**: 1470–1476

Wang J, Gao G and Goldfarb M (1994) Fibroblast growth factor receptors have different signalling and mitogenic potentials. *Mol Cell Biol* **14**: 181–188

Werner S, Shuhn D, Duan R, Devries C, Peters KG, Johnson DE. and Williams LT (1992) Differential splicing in the extracellular domain of fibroblast growth factor receptor 1 generates receptor variants with different ligand binding specificities. *Mol Cell Biol* **12**: 82–88

Yamaguchi F, Saya H, Bruner JM and Morrison RS (1994) Differential expression of two fibroblast growth factor receptor genes is associated with malignant progression in human astrocytomas. *Proc Natl Acad Sci USA* **91**: 484–488

Yan G, McBride G and McKeehan WL (1993) Exon skipping causes alteration of the COOH-terminus and deletion of the phospholipase C interaction site in the FGFR-2 kinase in normal prostate epithelial cells. *Biochem Biophys Res Commun* **194**: 512–518

© Cancer Research Campaign 1997